# Long-term sucrose solution consumption causes metabolic alterations and affects hepatic oxidative stress in Wistar rats

**DOI:** 10.1242/bio.047282

**Published:** 2020-02-28

**Authors:** Ellen Mayara Souza Cruz, Juliana Maria Bitencourt de Morais, Carlos Vinícius Dalto da Rosa, Mellina da Silva Simões, Jurandir Fernando Comar, Luiz Gustavo de Almeida Chuffa, Fábio Rodrigues Ferreira Seiva

**Affiliations:** 1 Department of Biology, Biological Science Center, Universidade Estadual do Norte do Paraná – UENP, Luiz Meneghel Campus, Bandeirantes, 8630-000 Paraná, Brazil; 2 Department of Biochemistry, Universidade Estadual de Maringá – UEM, Maringá, 87020-900 Paraná, Brazil; 3 Department of Anatomy, Institute of Biosciences of Botucatu, Universidade Estadual Paulista - UNESP, Botucatu, 18618-689 São Paulo, Brazil; 4 Post Graduation Program of Experimental Pathology, Department of Pathology, Universidade Estadual de Londrina – UEL, 86057-970 Paraná, Brazil

**Keywords:** Sucrose solution, Metabolic syndrome, Hepatic tissue, Oxidative stress

## Abstract

As the number of overweight and obese people has risen in recent years, there has been a parallel increase in the number of people with metabolic syndrome, diabetes and non-alcoholic fatty liver disease. The consumption of artificially sweetened beverages contributes to these epidemics. This study investigated the long-term effects of ingestion of a 40% sucrose solution on serum and hepatic parameters in male Wistar rats (*Rattus norvegicus*). After 180 days, the glycemic response, lipid profile and hepatic oxidative stress were compared to those of rats maintained on water. Sucrose ingestion led to higher body weight, increased fat deposits, reduced voluntary food intake and reduced feeding efficiency. Rats that received sucrose solution showed early signs of glucose intolerance and insulin resistance, such as hyperinsulinemia. Serum triacylglycerol (TG), very-low density lipoprotein (VLDL), cholesterol, ALT and AST levels increased after sucrose consumption. Elevated malondialdehyde and superoxide dismutase (SOD) levels and reduced glutathione levels characterize the hepatic oxidative stress due to sucrose ingestion. Liver sample histology showed vacuolar traces and increased fibrotic tissue. Our data showed the harmful effects of chronic consumption of sucrose solution, which can cause alterations that are found frequently in obesity, glucose intolerance and non-alcoholic hepatic disease, characteristics of metabolic syndrome.

## Abstract (precis)

**Summary:** Sucrose consumption in liquid form predisposes rats to obesity, alters glycemic and lipid profiles, and impairs hepatic oxidative metabolism; hence, we caution against excessive consumption of artificially-sweetened beverages.

## INTRODUCTION

During the past few decades, a dramatic rise in the overweight and obese population has become a global epidemic (Engin, 2017). Generally, an excess of energy intake over energy expenditure leads to obesity development, but genetics, medical conditions and lifestyle are factors that should not be ruled out (Skolnik and Ryan, 2014). Obesity can aggravate: (1) insulin resistance (IR); (2) diabetes mellitus type 2 (DM2); (3) dyslipidemia; and (4) accumulation of visceral fat. When these factors are combined, they characterize metabolic syndrome (MS), which in turn is associated with non-alcoholic fatty liver disease (NAFLD) (Caixas et al., 2014; Simopoulos, 2013).

Consumption of high-calorie, processed foods predisposes an individual to metabolic syndrome and hepatic disorders (Lustig et al., 2012; Stenvinkel, 2015). The consumption of artificially-sweetened beverages is also increasing in parallel with the prevalence of obesity and type 2 diabetes (Aydin et al., 2014; Malik et al., 2010). Sucrose (a disaccharide composed of glucose and fructose), or high-fructose corn syrup, containing about 50% to 55% fructose, are commonly added to artificially-sweetened beverages (Jensen et al., 2018). An important point that deserves attention is that liquid and solid meals differ in terms of energy gained, appetite and food intake responses (DiMeglio and Mattes, 2000; Pan and Hu, 2011; Stull et al., 2008). Many studies designed to evaluate the effects of sucrose and fructose use hypercaloric diets supplemented with lipids, carbohydrates, or both, in solid meals of animals from different species or strains (Abe et al., 2019; Devan et al., 2018; Jimoh et al., 2018; Lima et al., 2016; Sousa et al., 2018; Sun et al., 2018; Xu et al., 2019; Zhang et al., 2016). Few studies have investigated the effects of long-term (>3 months) consumption of sucrose solution versus drinking water in Wistar rats (*Rattus norvegicus*) (Aguilera et al., 2004; Chen et al., 2011; El Hafidi et al., 2001; Kawasaki et al., 2005; Masek et al., 2017). Some of these studies showed higher glycaemia and body weight caused by sucrose consumption, but others did not; furthermore, among these studies only Aguilera et al. (2004) evaluated lipid serum parameters, so more research might contribute to the understanding of the effects of liquid sucrose consumption.

Postprandial glucose disposal depends on normal liver function, and the composition of ingested carbohydrates influences hepatic glucose metabolism. Rats fed 10% sucrose solution developed hepatic steatosis and had impaired hepatic free radical defenses (Armutcu et al., 2005). Sucrose consumption causes fat accumulation in the liver, and subsequent development of hepatic insulin resistance, an independent risk factor for NAFLD (DiNicolantonio et al., 2015). The hyperinsulinemia due to insulin resistance increases precursors for hepatic fibrosis, reduces β-oxidation of fatty acids, and increases the generation of free radicals (Li et al., 2017; Paradis et al., 2001; Sun et al., 2018). The harmful effects of sugar-enriched diets and the association between sugar and NAFLD were comprehensively reviewed by Wong et al. (2016) and Jensen et al. (2018), respectively.

Because the effects of consuming sucrose solution are still being debated, and because the liver plays a pivotal role in glucose and lipid metabolism, we investigated the effects of long-term liquid sucrose consumption on nutritional, morphometric, serum biochemical markers and hepatic oxidative stress parameters, which may draw attention to the effects of excessive consumption of artificially sweetened beverages.

## RESULTS

**Fig. 1.**
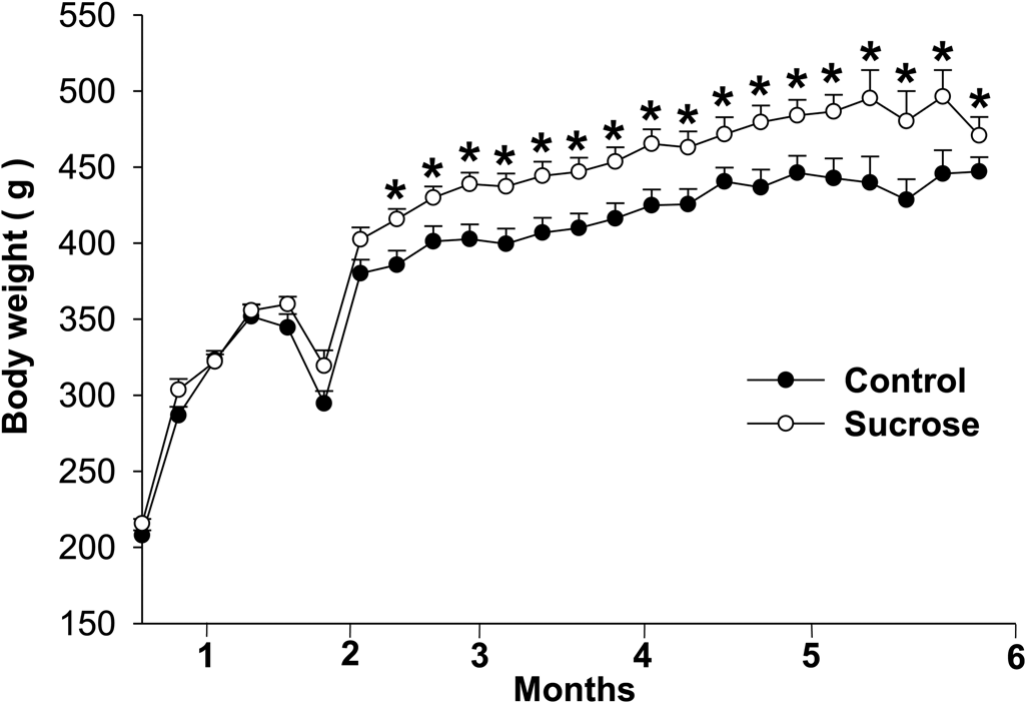
**Body-weight gain of rats receiving filtered water (Control) and Sucrose solution.** Data were expressed as mean±SE (*n*=6 in each group), and analyzed by Student's *t*-test; *P*≤5%.

Initial body weight was not statistically different between groups and was similar until the seventh week (Fig. 1). Animals in both groups had a higher final body weight compared to their initial body weight (*P*<0.001). After 180 days, the animals receiving sucrose solution had a higher final body weight than control animals (*P*=0.01). Food consumption (*P*<0.001), voluntary food intake (*P*<0.001) and feed efficiency (*P*<0.01) were lower in the sucrose group but liquid consumption (*P*<0.01), and energy intake (*P*<0.001) were higher. BMI, Lee index and abdominal circumference were similar, but the sucrose group had longer body length than the control group (*P*<0.01) (Table 1).

**Table 1.**
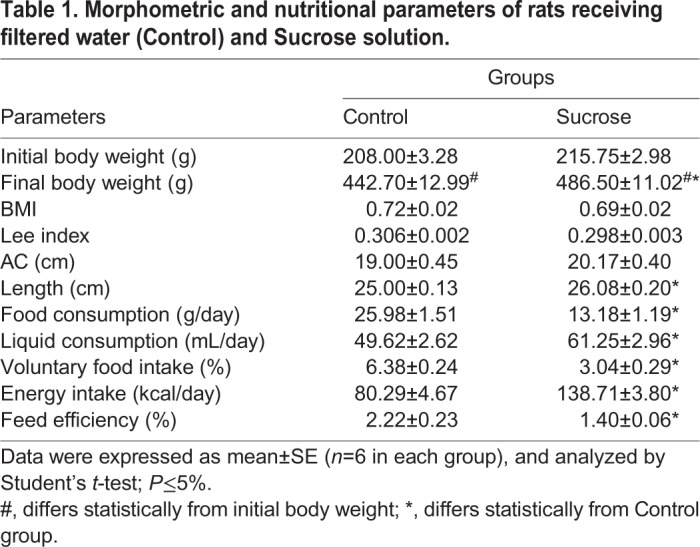
**Morphometric and nutritional parameters of rats receiving filtered water (Control) and Sucrose solution.**

**Fig. 2.**
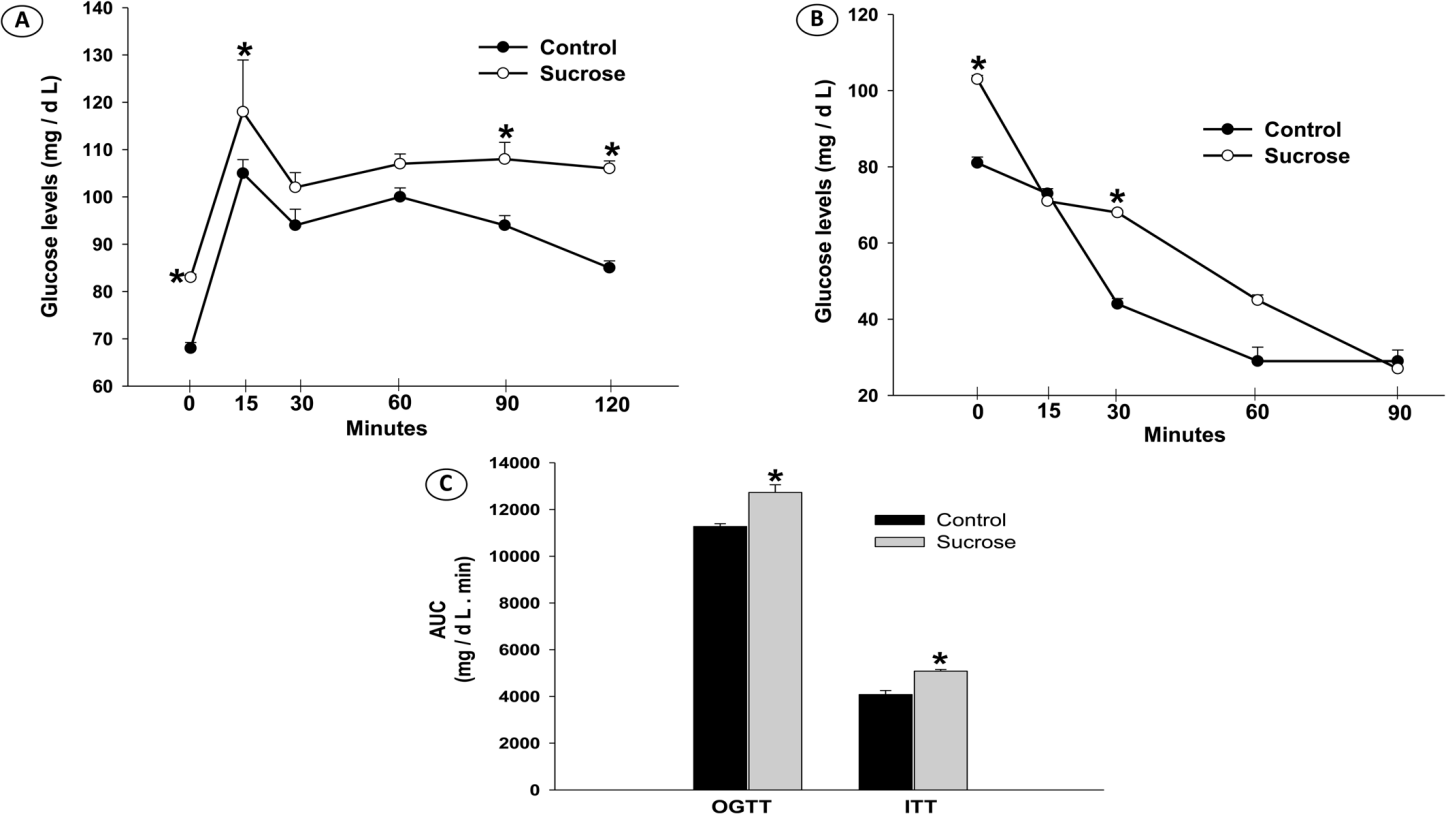
**(A) Oral glucose tolerance test; (B) insulin tolerance test; (C) AUC results of rats receiving filtered water (Control) and Sucrose solution.** Data expressed as mean±SE (*n*=6 in each group), and analyzed by paired *t*-test; P≤5%.

During oral glucose tolerance tests (OGTT), basal glucose levels were higher in the sucrose group (*P*<0.001) (Fig. 2A). After glucose injection, glycemia remained elevated at 90 min (*P*=0.018) and 120 min (*P*<0.001). The insulin tolerance test (ITT) results showed elevated basal glucose levels in sucrose-fed animals (*P*<0.001). In both groups, there was a reduction in glycemia in response to insulin injection; however, animals that consumed sucrose solution showed a delayed decrease in glucose concentrations (Fig. 2B). After 30 min, the glucose levels remained high in the sucrose group (*P*<0.001) (Fig. 2B). The area under the curve (AUC) – both OGTT-AUC and ITT-AUC – were elevated in the sucrose group (*P*<0.001) (Fig. 2C). The sucrose group had elevated fasting insulin (*P*=0.018), Homeostatic Model Assessment for Insulin Resistance (HOMA-IR) (*P*<0.001) and TyG index (*P*<0.001) values, and markedly reduced quantitative insulin check index (QUICKI) (*P*=0.002) and hepatic insulin sensitivity (HIS) (*P*=0.003) indices (Table 2).

**Table 2.**
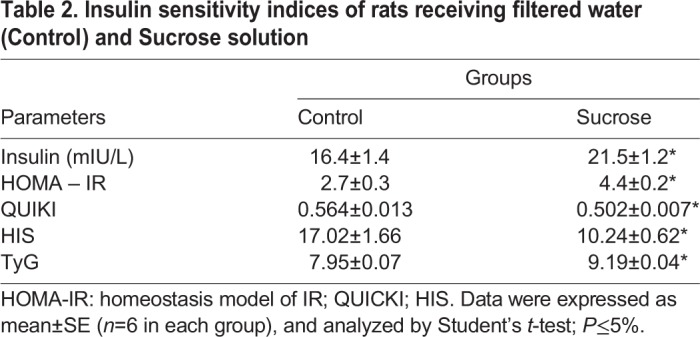
**Insulin sensitivity indices of rats receiving filtered water (**C**ontrol) and **S**ucrose solution**

**Fig. 3.**
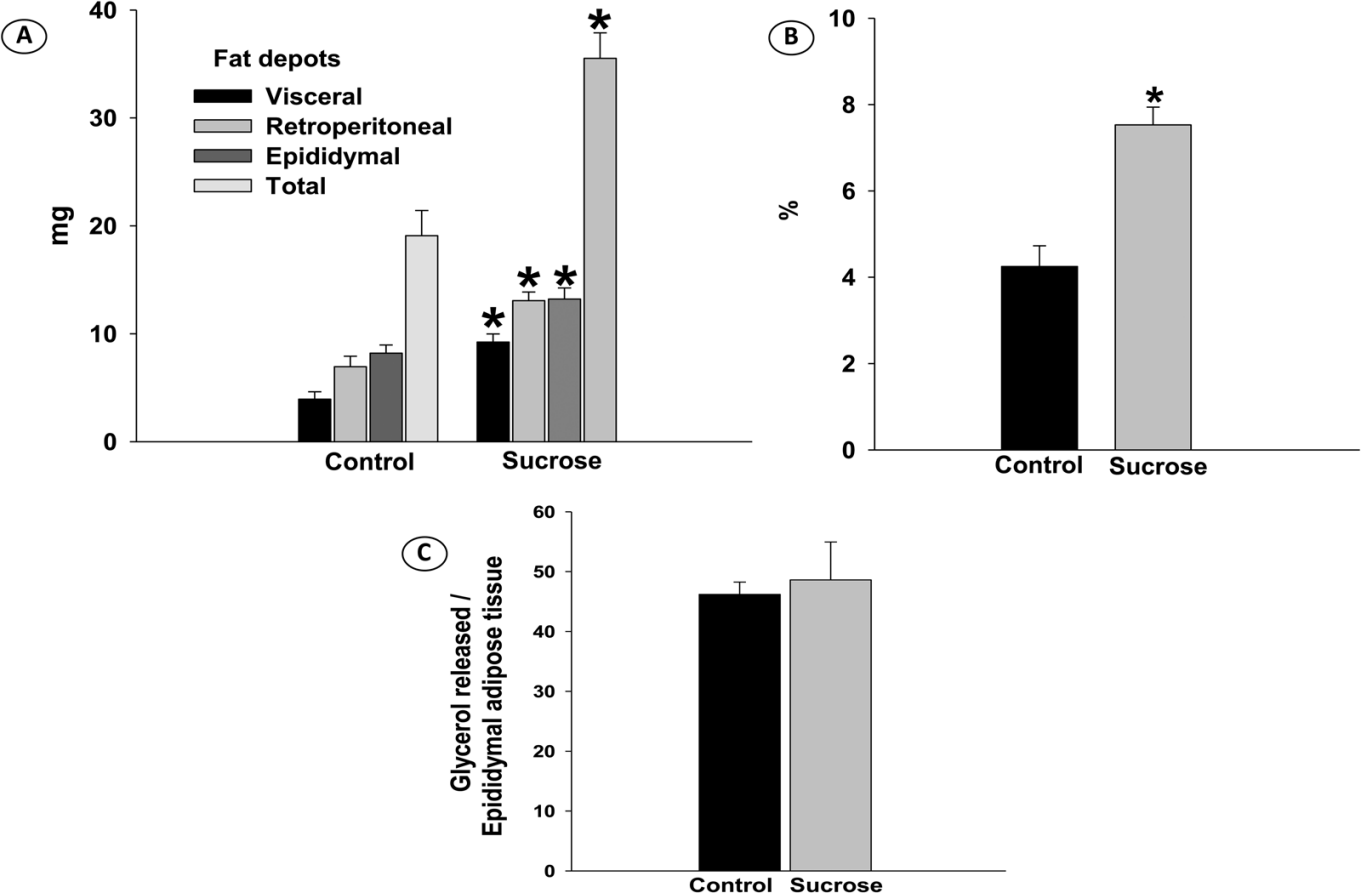
**(A) Fat depots, (B) adiposity index, (C) lipolysis basal rate of rats receiving filtered water (Control) and Sucrose solution.** Data were expressed as mean±SE (*n*=6 in each group), and analyzed by Student's *t*-test; *P*≤5%.

Sucrose consumption caused considerable increased visceral (*P*<0.001), retroperitoneal (*P*<0.001), epididymal (*P*=0.02) fat depots and adiposity index (*P*<0.001) (Fig. 3A,B). The basal lipolysis rate in the control and sucrose groups were not statistically different (Fig. 3C).

**Fig. 4.**
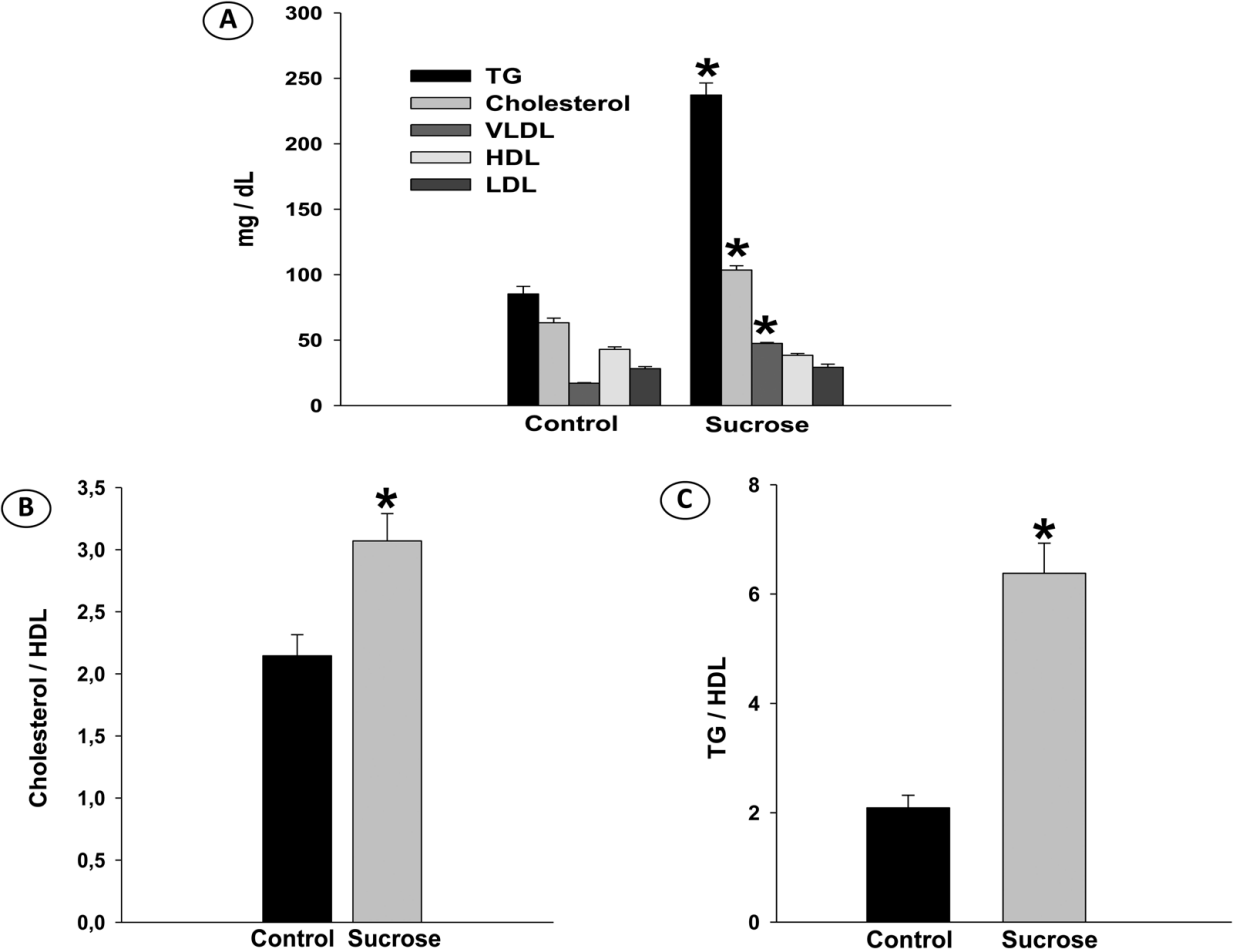
**(A) Serum lipid profile, (B) cholesterol/HDL ratio, (C) TG/HDL ratio of rats receiving filtered water (Control) and Sucrose solution.** Data were expressed as mean±SE (*n*=6 in each group), and analyzed by Student's *t*-test; *P*≤5%.

Sucrose solution consumption markedly increased serum TG (*P*<0.001), cholesterol (*P*<0.01) and VLDL (*P*<0.001) levels (Fig. 4A), thus supporting the dyslipidemia. The cardiovascular indices were higher in the sucrose group, TG/HDL (*P*<0.001) and TC/HDL (*P*<0.01) (Fig. 4B,C).

Sucrose ingestion elevated the alanine transaminase (ALT) (*P*<0.01) and aspartate transaminase (AST) (*P*<0.001) levels significantly. TG levels were higher in the livers of animals from the sucrose group (*P*=0.04), but cholesterol and glycogen levels were not statistically different (Table 3). The oxidative stress (OS) marker, Malondialdehyde (MDA), was elevated in rats consuming sucrose solution and these animals showed reduction of reduced glutathione (GSH), oxidized glutathione (GSSG), GSH+2× GSSG ratio (*P*<0.001 for all the referred parameters) and SOD activity (*P*<0.01) (Table 3).

**Table 3.**
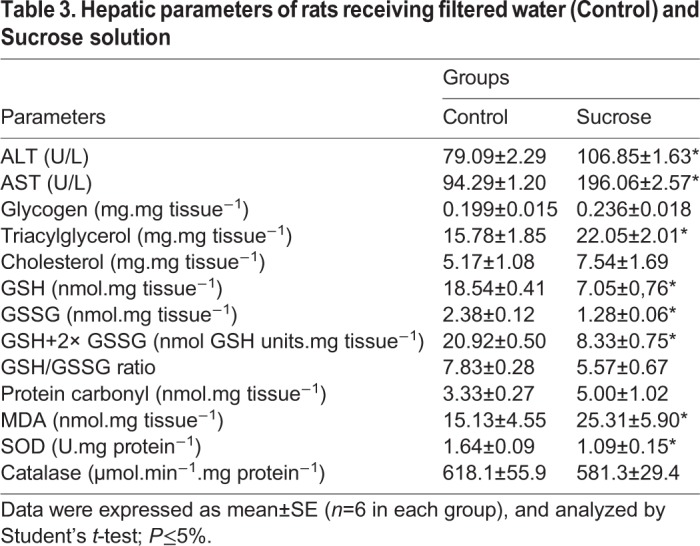
**Hepatic parameters of rats receiving filtered water (Control) and Sucrose solution**

**Fig. 5.**
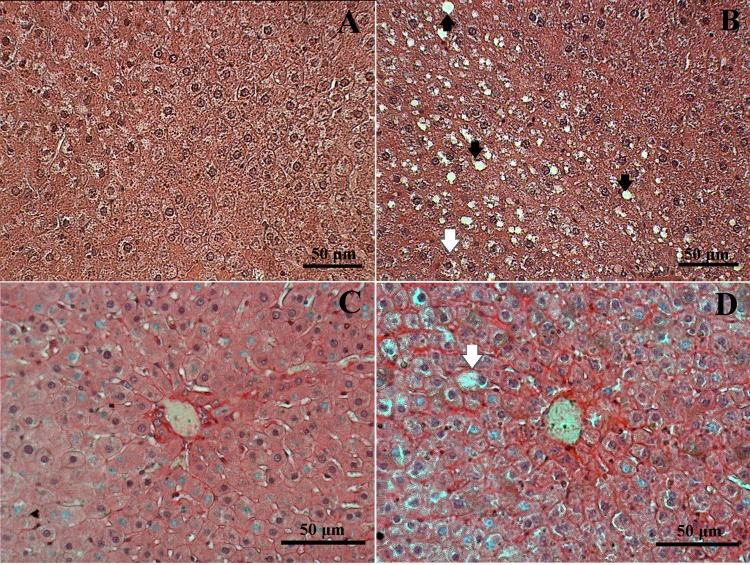
**Photomicrograph of section of H&E stained liver of rats receiving filtered water (Control) (A) and Sucrose solution (B).** Picrosirius Red stained section of liver of rats receiving filtered water (Control) (C) and Sucrose solution (D). The red stain in C and D indicates collagen. Black arrows indicate hepatocyte vacuolation, white arrows indicate hepatocyte ballooning.

Although not statistically different, the hepatocyte numbers were lower, while the hepatocyte size was increased in the sucrose group (Table 4). The sucrose group also showed higher hepatocyte ballooning and vacuolization within hepatocytes (Fig. 5B) compared to the control group (Fig. 5A). The collagen content in the hepatic parenchyma was significantly higher in the sucrose group and more concentrated in the perisinusoidal and pericentral regions (Fig. 5D).

**Table 4.**
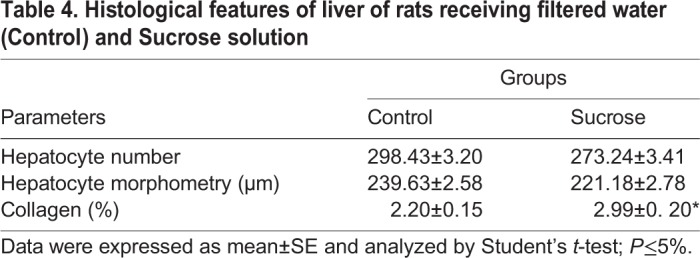
**Histological features of liver of rats receiving filtered water (Control) and Sucrose solution**

## DISCUSSION

Metabolic syndrome accompanied by insulin resistance, impaired glucose tolerance and dyslipidemia are closely associated with obesity, which in turn is related to excess dietary energy intake. Both metabolic syndrome and obesity are also related to hepatic disorders, including NAFLD (Rahman et al., 2017). In this study we confirmed the obesogenic effect of liquid sucrose consumption during a relative short period, i.e. after 8 weeks of disaccharide consumption, and the results are similar to those caused by high-fat diet consumption (Jimoh et al., 2018). The increased final body weight in the sucrose group was associated with increased fat depots and increased adiposity index. The animals consuming sucrose showed decreased food consumption and voluntary food intake, due to the high energy content of sucrose solution. Furthermore, sucrose ingestion diminished feed efficiency, suggesting that sucrose metabolism alters processes involved in the regulation of body weight and exhibits adverse nutritional effects. Morphometric parameters were not influenced by sucrose ingestion, similar to what was reported by Malafaia et al. (2013).

6 months after receiving the sucrose solution, rats exhibited similar glycemia. Rodrigues et al. (2016) showed no changes in glucose levels in rats fed with enriched fructose diet. Furthermore, normal fasting blood glucose levels were observed in subjects with diabetes (Bartoli et al., 2011). Other studies using sucrose-supplemented diet showed elevation (Chen et al., 2011), maintenance (Dutta et al., 2001) or reduction of glycemia (Del Toro-Equihua et al., 2016). Rats have stable energy metabolism, thus showing resistance to the development of full metabolic syndrome features with sucrose supplementation alone (Sun et al., 2018). In short, conflicting results are reported when fasting glucose alone is considered (Campbell et al., 2017). Hence, we conducted OGTT and ITT for an accurate assessment of the effects of sucrose solution consumption. Our results showed impaired glucose utilization in animals receiving sucrose, an early hallmark of type 2 diabetes mellitus, and obesity. The higher area under the curve corroborates these results and reflects those of Rahman et al. (2017), who demonstrated that high energy intake led to glucose intolerance. However, there is an important difference; animals from the Rahman et al. (2017) study were fed a high-carbohydrate and high-fat solid diet. Therefore, we emphasize that excessive consumption of sucrose-enriched beverages is as harmful as excessive high-carbohydrate and high-fat solid diet ingestion, considering the availability of glucose. Although the animals are euglycemic, glucose intolerance and insulin resistance are confirmed (Table 2). Liquid sucrose ingestion causes hyperinsulinemia and alters insulin sensitivity indices. Altogether, these data support the presence of insulin resistance due to sucrose solution consumption. These findings suggest that sucrose ingestion alters both pancreatic insulin secretion and peripheral tissues’ insulin sensitivity.

The hormone insulin is necessary for fat metabolism and both lipogenesis and lipolysis are critical pathways involved in balancing adipose tissue lipid content. The sucrose group showed marked elevation of fat deposition and adiposity index, supporting the earlier observations by Malafaia et al. (2013). Increased levels of glucose and fructose serve as acetyl-CoA sources for conversion to fatty acids for storage in hepatic and adipose tissues, thus contributing to NAFLD and obesity, respectively (Sanders and Griffin, 2016). Increased fat depots in the sucrose group could be due to higher caloric intake from the diet (Castro et al., 2017). Insulin resistance is known to increase adipose tissue lipolysis, although reduced fatty acid oxidation was documented in obesity and was associated with TG accumulation (Bouanane et al., 2010).

There are distinct causes of insulin resistance. For instance, GLUT4 is responsible for glucose transport in adipocytes, and altered expression might be related to intrinsic IR. Mice with GLUT4 deletion show insulin resistance, while GLUT4 overexpression improves glucose handling. In both cases, the fatty acid synthesis, termed *de novo* lipogenesis (DNL) is under the regulation of carbohydrate response element binding protein (ChREBP) (Vijayakumar et al., 2017) and sterol regulatory element binding protein 1C (SREBP1c) (Alves-Bezerra and Cohen, 2017) such that DNL is regulated primarily at the transcriptional level. Both transcriptional factors are activated by increased insulin signaling and increased glucose concentrations, which could partly explain our results. Further investigations are ongoing to test this hypothesis.

Dyslipidemia is a common feature in subjects with MS or DM2, thus reinforcing the close relationship between MS or DM2 and NAFLD (Sanders and Griffin, 2016). In hepatocytes, fructose, in contrast to glucose, undergoes alternative metabolism involving a highly specific enzyme, fructokinase (E.C. 2.7.1.4). This alternative step bypasses the PFK-1 regulatory role, to produce triose phosphates for DNL and interferes with the hepatic insulin signaling (Lim et al., 2010). In our experiment, the blood collection was from animals in a fasted state, and so the sole source for TG was the liver. Sucrose solution consumption induces hepatic TG synthesis, which explains the higher VLDL concentrations. Decreased endothelial cell lipoprotein lipase activity was associated with low TG clearance in fructose-fed rats (Nakagawa et al., 2006). The dyslipidemic profile seen in our rats was also shown by Oliveira Crege et al. (2016) and Wan et al. (2017) in studies of rats that consumed high-fat, and high-fat, high-cholesterol diets, respectively. The hypercholesterolemia and hypertriglyceridemia in the sucrose group indicated that sucrose solution consumption influences cardiovascular risk factors as well; such alterations may be associated with the fructose component of the solution. Nakagawa et al. (2006) showed that fructose, but not glucose, is related to MS. In brief, dyslipidemia caused by sucrose consumption is associated with increased risk for hepatic and cardiac disorders, and altered insulin-mediated metabolic responses; features consistent with MS.

The increased ALT and AST levels after sucrose consumption, indicate hepatocyte injury, thus corroborating data from rats fed solid sucrose-enriched diet (Xu et al., 2019). In the skeletal muscle of animals and humans, TG accumulation is an important contributor to muscle IR (Pan et al., 1997). This ectopic accumulation of lipids affects the liver, and contributes to NAFLD (Monteillet et al., 2018). We speculate an association of higher hepatic TG with reduced HIS, contributing to hyperinsulinemia in our rats. The higher content of fat depots impairs the peripheral insulin action (Oliveira Crege et al., 2016). Interestingly, some studies have discussed the accumulation of liver TG as a potential protective mechanism (Tilg and Moschen, 2010; Unger and Scherer, 2010).

Animals from the sucrose group displayed evidences of hepatic metabolic imbalance, such as reduced glutathione and SOD (Table 3). Moreover, increased MDA and decreased GSH/GSSG and GSH+2x GSSG ratios are also indicative of hepatic metabolic dysfunction, culminating in OS, which could be due to impaired ROS scavenging system and ROS production (Sa-Nakanishi et al., 2018), thus suggesting that rat livers from the sucrose group failed to maintain the redox state. An association between carbohydrate consumption and OS is seen frequently in the literature (Ecker et al., 2017; Kosuru and Singh, 2017). Gregersen et al. (2012) showed reduced total antioxidant capacity in the blood shortly after a high carbohydrate meal. Similarly, our data show that even without fasting hyperglycemia, glucose intolerance, and IR interfere with hepatic oxidative parameters. However, it cannot be ruled out that a higher hepatic TG content may be associated with elevated OS in sucrose-fed rats.

Rats from the sucrose group showed morphological alterations in the hepatic parenchyma. Sugar ingestion initiates structural changes, such as a reduction in hepatocyte number and increased hepatocyte size, characteristics related to hepatocyte ballooning, a frequent feature of the damaged liver (Corona-Perez et al., 2017). We also observed higher vacuolation in the sucrose group, which is related to increased cellular deposits and cellular damage (Nayak et al., 1996). Moreover, we observed the presence of collagen in the liver of sucrose-fed animals, and the mild fibrosis occurred mainly in the perisinusoidal region and pericentral zone, relating to NAFLD (Kleiner et al., 2005). The higher levels of transaminases found in our study suggest disruption of the liver function (Kaswala et al., 2016). Corona-Perez et al. (2017) showed similar results with 8 weeks of sucrose supplementation, classifying their results as moderate grade fibrosis. In contrast, Masek et al. (2017) showed no evidence of liver fibrosis after 20 weeks of sucrose supplementation, despite higher lipid content.

Lastly, we discuss two limitations of this study: first, we did not investigate the molecular mechanisms underlying these alterations; second, since sucrose solution reduced food consumption by >50%, it could have resulted in nutritional imbalances in the animals. Future studies are required to address the above findings.

In conclusion, consumption of 40% sucrose solution for 180 days caused elevation of fat depots, altered glycemic and lipid profiles. Hepatic tissue showed elevated oxidative stress markers and morphological alterations, hallmarks of NAFLD. Altogether, sucrose-containing beverages could be obesogenic, and the physical form of the ingested carbohydrates is relevant for understanding the global health problems occurring over the past decades.

## MATERIALS AND METHODS

### Animals and experimental protocol

The experimental design and the analysis were conducted following the ethical principles for animal research established by the Brazilian Council for Control of Animal Experimentation, and approved by the Animal Ethics Committee of North of Parana State University, UENP, Brazil (protocol number: CEUA 05/2017).

Twelve male Wistar rats, 60 days old, were housed in an environmentally-controlled clean-air room, under standard temperature (22±3°C), 12 h light-and-dark cycles and relative humidity of 60±5%. The animals were randomly assigned into two groups (*n*=6). Both groups received standard chow (NuvilabCR1^®^, Nuvilab, Brazil) *ad libitum.* Controls (C), received filtered water, and the Sucrose (S) treatment rats received 40% sucrose solution prepared daily. This treatment continued for 180 days.

### Nutritional and morphometric parameters

Every week body weight, food and liquid consumption were measured between 9–10 am, and the difference between the total and the leftover food and drinking solution calculated. Food and liquid intake, and caloric values of chow (3.09 kcal/g) (Lima et al., 2016) and sucrose solution (1.6 kcal/mL), were used to obtain the following parameters: total energy intake (EI, kcal/day), feed efficiency (FE, %) and, voluntary food intake (VFI, %) (Seiva et al., 2012). At the end of the experimental period, animals under anesthesia had their body weight (BW), and body length (BL) determined to calculate body mass index (BMI) and Lee index [cube root of body weight (g)/nose-to-anus length (cm)] (Chuffa and Seiva, 2013). Abdominal circumference was also measured.

### Oral glucose and insulin tolerance tests

5 and 2 days before the end of the experiment, rats were submitted to oral glucose tolerance test (OGTT) and insulin tolerance test (ITT), respectively, and blood samples were collected for insulin quantification. For the OGTT, rats were deprived of food for 6 h, and were administered 2 g/kg body weight of glucose as a 20% aqueous solution via oral gavage. Blood samples were obtained from the tail vein before and at 15, 30, 60, 90 and 120 min after glucose administration. For the ITT, rats were injected intra-peritoneally with 1 U/kg of BW of regular human insulin (Humulin™ Eli Lilly, São Paulo, Brazil) and blood samples collected before and at 15, 30, 60 and 90 min after insulin administration. The trapezoidal rule was used to determine the AUC for OGTT and ITT (Antunes et al., 2016). Blood glucose levels were measured using an automated glucose analyzer (Accu-Chek Active, Roche^®^, SP, Brazil). Serum insulin quantification was performed using an enzyme-linked immunosorbent assay kit (Mouse Insulin ELISA kit, Thermo Fisher Scientific, USA). The insulin sensitivity indices, such as homeostasis model assessment of insulin resistance (HOMA IR=fasting insulin×fasting glucose/22.5) (Antunes et al., 2016); quantitative insulin sensitivity check index [QUICKI=1/(log insulin concentration+log blood glucose concentration)] (Bowe et al., 2014); the product of fasting plasma glucose and triglyceride [TyG index=ln (fasting triacylglycerol×fasting glucose/2)] (Lee et al., 2018); hepatic insulin sensitivity (HIS=1000/fasting insulin×fasting glucose) (Matsuda and DeFronzo, 1999) were calculated. HIS assumes that higher values of the product of glucose and insulin are inversely related to the capacity of the liver to respond to insulin.

### Biological material collection

At the end of the experimental protocol, fasting rats were euthanized by barbiturate overdose (Zatroch et al., 2017). Blood samples were collected via cardiac puncture, placed into centrifuge tubes, allowed to clot and centrifuged at 5000 rpm for 10 min. Serum aliquots were frozen at −80°C for the following analyses: ALT, AST, TG, total cholesterol (TC), and high-density lipoprotein (HDL) were determined using commercial enzymatic methods (CELM diagnosis, São Paulo, Brazil). The concentration of VLDL and low-density lipoprotein was measured as described elsewhere (Pinafo et al., 2019). The values from the above determinations were used to calculate the cardiometabolic risk indices as follows, CR1=TC/HDL (Kosuru and Singh, 2017) and CR2=TG/HDL (Wang et al., 2017).

White adipose tissue depots, retroperitoneal, epididymal and visceral were dissected rapidly and weighed, and the data were used to calculate the adiposity index (%) (Seiva et al., 2011). Epididymal adipose tissue portions (50 mg) were collected and incubated for 1 h in an appropriate buffer at 37°C, and aliquots were heated to 70°C to inactivate enzyme. Glycerol levels were measured using a commercial kit, and the ratio of the concentration of glycerol and epididymal weight was determined as the basal lipolytic index (Rodrigues et al., 2016). The liver was dissected and weighed, and liver samples were stored at −80°C for the determination of hepatic oxidative stress and glycogen content. The left lobe of the liver was collected and used for histological preparations.

### Hepatic parameters

For preparing the liver homogenate, the frozen tissue was homogenized in a Potter-Elvehjem homogenizer with 10-volumes of ice-cold 0.1 M potassium phosphate buffer (pH 7.4). The homogenate was centrifuged at 10,000 rpm for 15 min, and the supernatant used as the soluble fraction. Protein carbonyl groups were measured spectrophotometrically using 2,4-dinitrophenylhydrazine (Levine et al., 1990). The levels of protein carbonyl groups were calculated using the molar extinction coefficient (*ε*) of 2.20×104 M^−1^·cm^−1^. MDA was measured by the thiobarbituric acid-reactive substances assay (Buege, 1978). The levels of glutathione, reduced (GSH) and oxidized (GSSG) were measured with a spectrofluorimetric method (excitation at 350 nm and emission at 420 nm) using the o-phthalaldehyde assay as described earlier (Hissin and Hilf, 1976). The catalase activity using H_2_O_2_ as substrate was determined by measuring the change in absorbance at 240 nm (Aebi, 1974), and the results were calculated using the molar extinction coefficient (*ε*) of 9.6×10^−3^ M^−1^·cm^−1^. The activity of SOD was estimated by its capacity to inhibit pyrogallol autoxidation in the alkaline medium at 420 nm (Marklund and Marklund, 1974). One SOD unit is equal to the quantity of enzyme that causes 50% inhibition, and the results were expressed as units·mg protein^−1^.

Approximately 200 mg of hepatic tissue was homogenized with 0.01 M sodium phosphate buffer (pH 7.4), using an L-beader cell disrupter with 3 mm zirconium beads (3720 rpm, 2 min). Later, the homogenate was centrifuged at 10,000 rpm for 15 min at 4°C, and the supernatant collected for quantification of total glycogen content by the anthrone assay (Roe and Dailey, 1966).

### Liver processing and histological analysis

After 24 h of fixation in Bouin's solution, the left lobe of the liver was processed for histology (dehydrated, diaphanized, and paraffin-embedded) and 5-µm thick semi-serial sections were obtained. These sections were stained with either Hematoxylin and Eosin (H&E) (for morphometric analysis of hepatocytes), or Picrosirius Red counter-stained with Hematoxylin (for collagen analysis).

The liver microphotographs were captured using a light microscope coupled to a high-resolution camera, with a 200× magnification in the area of the central vein. For the H&E stained slides, from each animal, 30 images were captured with an exact area of 0.216 mm^2^ each, excluding a fixed area around the central vein. The hepatocyte numbers in these images were counted, the cytoplasmic area of 100 hepatocytes/animal were measured, and the images analyzed using the program Image Pro Plus 4.5 software (Media Cybernetics, Maryland, USA).

We captured 30 images/animal with the Picrosirius Red-stained slides to evaluate the area marked for collagen. The area analyzed was the same from the H&E technique. The percentage area of collagen was obtained using the ImageJ FIJI software, based on the threshold from RGB stacks.

### Statistical analysis

Statistical comparisons were performed using the Sigma Plot software (version 11.0). The results are presented as the mean±SEM and are discussed considering *P*<5%. The results were analyzed using Student's *t*-test. For OGTT and ITT tests, glycaemia was measured at different times after the oral load of glucose. Differences between control and sucrose groups, within fixed time, were analyzed by repeated samples (paired) *t*-test.
